# pTuneos: *p*rioritizing *tu*mor *neo*antigens from next-generation *s*equencing data

**DOI:** 10.1186/s13073-019-0679-x

**Published:** 2019-10-30

**Authors:** Chi Zhou, Zhiting Wei, Zhanbing Zhang, Biyu Zhang, Chenyu Zhu, Ke Chen, Guohui Chuai, Sheng Qu, Lu Xie, Yong Gao, Qi Liu

**Affiliations:** 1 Department of Endocrinology & Metabolism, Shanghai Tenth People’s Hospital; Bioinformatics Department, School of Life Sciences and Technology, Tongji University, Shanghai, 200092 China; 2 Department of Ophthalmology, Ninghai First Hospital, Ninghai, 310000 Zhejiang China; 3 Shanghai Center for Bioinformation Technology, Shanghai, 201203 China; 4 Department of Digestive Oncology, Shanghai East Hospital, Tongji University, Shanghai, 200120 China

**Keywords:** Cancer neoantigen, Next-generation sequencing, Immune checkpoint blockade, Biomarker, Immunotherapy

## Abstract

**Background:**

Cancer neoantigens are expressed only in cancer cells and presented on the tumor cell surface in complex with major histocompatibility complex (MHC) class I proteins for recognition by cytotoxic T cells. Accurate and rapid identification of neoantigens play a pivotal role in cancer immunotherapy. Although several in silico tools for neoantigen prediction have been presented, limitations of these tools exist.

**Results:**

We developed *pTuneos*, a computational pipeline for *p*rioritizing *tu*mor *neo*antigens from next-generation *s*equencing data. We tested the performance of *pTuneos* on the melanoma cancer vaccine cohort data and tumor-infiltrating lymphocyte (TIL)-recognized neopeptide data. *pTuneos* is able to predict the MHC presentation and T cell recognition ability of the candidate neoantigens, and the actual immunogenicity of single-nucleotide variant (SNV)-based neopeptides considering their natural processing and presentation, surpassing the existing tools with a comprehensive and quantitative benchmark of their neoantigen prioritization performance and running time. *pTuneos* was further tested on The Cancer Genome Atlas (TCGA) cohort data as well as the melanoma and non-small cell lung cancer (NSCLC) cohort data undergoing checkpoint blockade immunotherapy. The overall neoantigen immunogenicity score proposed by *pTuneos* is demonstrated to be a powerful and pan-cancer marker for survival prediction compared to traditional well-established biomarkers.

**Conclusions:**

In summary, *pTuneos* provides the state-of-the-art one-stop and user-friendly solution for prioritizing SNV-based candidate neoepitopes, which could help to advance research on next-generation cancer immunotherapies and personalized cancer vaccines. *pTuneos* is available at https://github.com/bm2-lab/pTuneos, with a Docker version for quick deployment at https://cloud.docker.com/u/bm2lab/repository/docker/bm2lab/ptuneos.

## Background

Recent tumor immunotherapy studies demonstrated that exploiting a patient’s own immune system is an advanced strategy for eliminating cancer cells [1]. Tumor-specific neopeptides (so-called neoantigens), some of which could be presented on the tumor cell surface complexed with human leukocyte antigen (HLA) class I protein, play an important role in this process. The recognition of peptide-major histocompatibility complex (MHC)-I complexes by cytotoxic T cells can activate the T cell response [2, 3]. Moreover, the tumor mutation load and predicted neoantigen load are reported to strongly correlate with the clinical response to immune checkpoint inhibition in several cancer types [4–6].

Somatic mutations are largely heterogeneous across different cancers and different patients. Therefore, neoantigens must be identified and evaluated at a personalized level [7, 8]. In general, the prediction of neoantigens based on next-generation sequencing (NGS) data comprises three steps: (1) obtain a list of genomic somatic mutations from whole-exome sequence data and convert it into mutation-containing “neopeptides” of appropriate lengths, (2) predict the binding affinity between the peptides and patient-specific HLA alleles, and (3) evaluate the immunogenicity of the predicted peptides [9, 10]. To date, several in silico tools for single-nucleotide variant (SNV)-based candidate neoepitopes prediction have been described, including *pVAC-Seq* [11], *MuPeXI* [12], *TSNAD* [13], and *Neopepsee* [14]. *pVAC-Seq* and *TSNAD* focus on MHC-I binding affinity and implement filter-based strategies to obtain the final neopeptide without prioritization, which prevents its further clinical utilization. *MuPeXI* prioritizes the candidate peptide based on limited in vitro information. *Neopepsee* constructs a machine-learning model based on the immunogenicity features of the peptide to optimize the candidate neoepitope set. Among these tools, only *Neopepsee* provides a learning-based measurement of neoepitopes, but issues remain to be overcame: (1) features used in *Neopepsee* might be irrelevant and difficult to interpret biologically, (2) the training data used in *Neopepsee* lack specificity as the peptides come from generic antigens rather than true noeantigens with experimental validation, and (3) the training data used in *Neopepsee* are highly imbalanced. Such training data may induce substantial bias in actual neoantigen identification. Furthermore, all the available tools are developed based on the data obtained from MHC multimer technology, which stimulates patient-derived T cells with a synthetic MHC-peptide complex, indicating that these tools are mainly designed to predict the ability of MHC presentation and T cell recognition of the candidate neopeptide in vitro. However, the actual immunogenicity of neoantigen in patient tumor might be influenced not only by the MHC presentation and T cell recognition, but also by many other endogenous factors including neopeptide cleavage probability, transporter associated with antigen processing (TAP) transport efficiency, peptide expression level, mutation allele fraction, and neoantigen cellular prevalence. None of the existing tools provides a quantitative and comprehensive metric to evaluate these characteristics and the immunogenicity of the naturally processed and presented neoantigen, which is the most challenging issue for clinical application of these tools.

Here, we present a novel computational strategy to address the abovementioned issues with its implementation. The program, called *pTuneos* (*p*rioritizing *tu*mor *neo*antigens from next-generation *s*equencing data), presents an efficient in silico tool to predict the immunogenicity of SNV-based neopeptides based on experimentally validated neoantigens, surpassing the existing tools with a comprehensive and quantitative benchmark of their neoantigen prioritization performance and running time. Together, *pTuneos* addresses the above challenges with the following advantages: (1) *pTuneos* firstly presented a learning-based framework, i.e., *Pre&RecNeo* to predict and prioritize neoepitopes recognized by T cell. This module can be applied to predict the MHC presentation and T cell recognition ability of the neoantigens, and it is suitable for the evaluation of the neopeptide in vitro immunogenicity. Then, *pTuneos* presented a novel neoepitope scoring schema, i.e., *RefinedNeo* to evaluate the naturally processed and presented neoepitope immunogenicity (defined as the *refined immunogenicity score*), which was demonstrated to successfully refine the neoepitope ranking list obtained by the *Pre&RecNeo* model and filters out those neoepitopes that could be recognized by T cell but could not be naturally processed and presented. This module can be applied to prioritize the in vivo immunogenicity of the peptides. (2) The refined immunogenicity score is demonstrated to be a powerful and pan-cancer marker for survival analysis compared to traditional well-established biomarkers on TCGA data. (3) The refined immunogenicity score is demonstrated to be leveraged to better predict survival in anti-CTLA-4-treated melanoma patients and anti-PD-1-treated lung cancer patients. (4) A quantitative evaluation measurement is presented to comprehensively evaluate the predicted neoantigen ranking result based on the golden standard data. (5) An efficient data synthesizing technique is applied to address the data imbalance issue for model training. (6) Multiple thread processing is implemented in *pTuneos* for running speed acceleration, and (7) *pTuneos* can be quickly installed and deployed with the Docker version at https://cloud.docker.com/u/bm2lab/repository/docker/bm2lab/pTuneos.

## Implementation

### Design of *pTuneos* pipeline

#### Data preprocessing

##### Processing of whole-genome or whole-exome sequencing (WGS/WES) data

Sequencing quality control was performed using Trimmomatic-0.36 [15] to trim the read below an average Phred score of 20 and cut out standard adapters. Reads were aligned to the human genome (hg38) using the Burrows-Wheeler Aligner version 0.7.12 [16]. A BAM file was sorted and produced with the Picard version 2.3.0 SortSam, and duplicate reads were marked and removed using the Picard tool MarkDuplicates. Base recalibration was performed with GATK version 3.8.0 [17] to reduce false-positive variant calls. SNV calls were performed with Mutect2 while indel calls were created utilizing GATK Mutect2 version 3.8.0 [18], Varscan2 [19], and Strelka2 [20]. All mutations with allelic fractions of less than 0.05 or coverage of less than 10× were excluded to eliminate false-positive sites. HLA alleles of each sample were inferred from trimmed WGS or WES data using OptiType [21] with default settings that could achieve HLA typing with ~ 97% accuracy.

##### Processing of RNA-seq data

Kallisto [22] was utilized to quantify the abundance of gene isoforms from the RNA-seq data. The reference transcriptome was downloaded from the Ensembl database for GRCh38 using Ensembl genome browser version 89. The data were indexed using the default read-length option of 100–200 bp in the RNA-seq data. The abundance of gene isoforms was calculated as transcripts per kilobase million.

#### Candidate neoepitope identification

##### Mutation annotation and peptide extraction

All the mutations were annotated with Ensembl Variant Effect Predictor [23] (VEP) to identify non-synonymous mutations, including SNVs and indels. For SNVs, the genomic change was directly applied to the proteome reference, leading to a 21-mer mutant peptide and a normal peptide, and the peptides were cut into 9–11-mer short peptides that match the length of the neoantigen. For indels, the mutant protein sequence was inferred by translating the mutant cDNA sequence and 9–11-mer short peptides were also produced.

##### Epitope prediction

Both mutant peptide binding affinity and normal peptide binding affinity were predicted between peptides and the (up to 6) patient-specific HLA alleles using NetMHCpan version 4.0 [24] in the binding affinity (BA) model. The percent rank score of binding affinity was obtained for neoantigen filtering because this metric is less biased than binding affinity when comparing binding between multiple HLA alleles. Neopeptides with a percent rank score greater than 2 were excluded to obtain candidate neoantigens that could be confidently presented by the MHC-I molecule.

#### Model building for MHC-presented and T cell-recognized neoepitope prediction

Currently, MHC multimer analysis is the most popular technology for detecting an antigen-specific T cell response [25]. Patient-derived T cells such as peripheral blood mononuclear cells were cultured in vitro, followed by stimulation of a synthetic peptide-MHC complex, and peptides that elicited T cell immunoreactivity were considered experimentally validated immunogenic neoepitopes. We considered the following five non-redundant features related with neoepitope presentation and recognition, including mutant peptide-MHC affinity percentile rank, the normal peptide-MHC affinity percentile rank, sequence similarity between the normal and mutant peptides, the peptide hydrophobicity score, and T cell recognition of the peptide-MHC complex. We performed feature engineering to scale every feature to the same range considering the biologic value.

##### Mutant and normal peptide percentile rank score

Percentile rank was used to measure MHC-I binding affinity instead of IC_50_ because this percentile rank is less biased than IC_50_ when comparing binding between multiple HLA alleles [26]. The percentile rank was scaled from 0 to 1 by a negative logistic function as a binding affinity score *L*(*x*), which is given by:

1 $$ L(x)=\frac{1}{1+{e}^{5\left(x-2\right)}} $$

This function gives a value approaching 0 for a high percentile rank, a midpoint at a percentile rank of 2, and a value of 1 for a low percentile rank. The constant 2 defines the inflection point, and it was selected as the recommended cutoff for possible peptide binding given by NetMHCpan. The function was applied to both the mutant peptide-MHC affinity percentile rank and the normal peptide-MHC affinity percentile rank, leading to a mutant peptide percentile rank score and a normal peptide percentile rank score.

##### Self-sequence similarity between normal and mutant peptides

Several studies demonstrated that sequence similarity is an important feature of immunogenicity [27]. Using the BLOSUM62 matrix, the amino acids at each position along the paired tumor and normal peptides were obtained as an aggregate similarity score, with higher scores indicating higher similarity. As these scores vary depending on the amino acid composition of the peptide tested, we performed a normalization by dividing the similarity score for a neoantigen compared with another peptide by the similarity score of the neoantigen tested against itself to produce self-similarity scores, which gave a value between 0 and 1, where a value of 1 indicates a perfect match.

##### Peptide hydrophobicity score

The hydrophobicity of amino acids at T cell receptor (TCR) contact residues is a strong hallmark of CD8+ T cell-mediated immunity [28]. We first collected all peptide MHCs with a positive T-cell response classified as the immunogenic peptide group and the nonimmunogenic self-peptide group, which represents cell surface ligand-eluted MHC-I self-peptides that were antigenically processed and MHC-bound from Immune Epitope Database (IEDB, www.iedb.org). Additional curation resulted in a final dataset with 5018 9–11-mer immunogenic peptides and 8227 9–11-mer nonimmunogenic peptides. Next, we constructed three eXtreme Gradient Boosting (XGBoost) algorithm-based machine-learning models to predict the probability of peptides recognized by T cells corresponding to 9-mer, 10-mer, and 11-mer peptides, respectively (Additional file 1: Figure S1. A). A 10-fold cross-validation reaches an area under the curve (AUC) score of 0.68, 0.77, and 0.77 corresponding to 9-mer, 10-mer, and 11-mer peptides (Additional file 1: Figure S1. B, C, D), respectively, which outperformed the model trained by the three-layer neuron network reported by Chowell et al. [28]. The output of each model represents the T cell recognition probability ranging from 0 to 1.

##### T cell recognition probability of the peptide-MHC complex

Early studies revealed that TCRs have relatively low affinities for their peptide-MHC ligands, making studies of TCR:pepMHC binding prediction difficult [29]. Recently, several methods measuring the T cell recognition probability of peptide MHCs were proposed based on a sequence comparison analysis [14, 30, 31]. Here, we used the computational model presented by Luksza et al. to calculate the T cell recognition probability. The model gives *R*, the probability that a neoantigen will be recognized by the TCR repertoire, by alignment with a set of peptides retrieved from Immune Epitope Database (IEDB). These peptides are linear epitopes from human infectious diseases that are positively recognized by T cells after class I MHC presentation. The model assumed that a neoantigen predicted to cross-react with a TCR from this pool of immunogenic peptides is a neoantigen that is more likely to be immunogenic itself, as members of the TCR repertoire both recognize a high number of presented antigens and have intrinsic biases in their generation probability. *R* is defined by a multistate thermodynamic model. In this model, sequence similarity is treated as a proxy for binding energy. To assess the sequence similarity between a neoantigen with peptide sequence s and an IEDB epitope *e*, a gapless alignment between the two sequences with a BLOSUM62 amino acid similarity matrix was computed and their alignment scores were denoted as |*s*,*e*|. Given these sequence similarities, for a given neoepitope with peptide sequence *s*, the probability that it will bind to a TCR specific to some epitope *e* from the IEDB pool was calculated as:

2 $$ R=Z{(k)}^{-1}\;\sum \limits_{e\in \mathrm{IEDB}}\exp\;\left(-k\;\left(a-|s,e|\right)\right) $$

where *a* represents the horizontal displacement of the binding curve, *k* sets the steepness of the curve at *a*, and

3 $$ Z(k)=1+\sum \limits_{e\in \mathrm{IEDB}}\exp\;\left(-k\;\left(a-|s,e|\right)\right) $$

which represents the partition function over the unbound state and the all-bound state. Here, *k* = 4.87 and *a* = 26, which were determined in the original study [31].

##### Collection of training data and testing data for model building

Training data were gathered from 16 studies relating to cancer immunotherapy (Additional file 2: Table S1). These studies assessed the immunogenicity of larger sets of neopeptides and published lists of neopeptides that did or did not elicit a T cell response in vitro. In 14 of 16 studies, both neopeptides and their corresponding unmutant peptides were retrieved. In the other two studies, some neopeptides resulted from genomic frameshift indels and their corresponding normal peptides were missing or partially missing, and therefore, we identified the most similar peptide by aligning the neopeptide to the reference human proteome with the BLOSUM62 amino acid similarity matrix. The human reference sequence proteome (release 89 based on genome GRCh38) was downloaded from Ensembl. The final training dataset included 2191 peptides that were experimentally tested, 84 of which could elicit a T cell response, resulting in 2107 negative samples and 84 positive samples. The testing dataset was obtained from Carreno et al. [32]. Nine of 21 tested peptides were immunogenic (Additional file 3: Table S2).

##### Handling data imbalance issue

As the training set was extremely imbalanced (84 vs 2107), the classifier trained on this kind of data would be biased; thus, the Synthetic Minority Over-sampling Technique (SMOTE) was applied to the dataset to address this problem. SMOTE [33] is an over-sampling approach in which the minority class is over-sampled by creating “synthetic” examples rather than by over-sampling with replacements and the minority class is over-sampled by taking each minority class sample and introducing synthetic examples along the line segments joining any/all of the *k* minority class nearest neighbors. We performed this process utilizing python package *imblearn* with parameters *k* = 4 and kind = “borderline1”, leading to a balanced dataset for model training.

##### Model building

Finally, we constructed two machine-learning classifiers: eXtreme Gradient Boosting [34] (XGBoost) and random forest (RF). XGBoost was built using the xgboost package, and the learning rate, maximum tree depth, and other hyper-parameters were tuned by built-in cross-validation coupled with a parameter grid search method. RF was built using the sklearn ensemble package by adjusting the option of using out-of-bag samples to estimate the generalization accuracy (oob_score) to true. The performance of the two classifiers was measured identically by 10-fold cross-validation on the training set and testing set and reached a training AUC of 0.987 and 0.998 and a testing AUC of 0.654 and 0.833, respectively. Therefore, RF was selected as the final classifier in our model.

#### Score scheme for neoepitope immunogenicity prioritizing

The model presented in the former section could predict the MHC presentation and T cell recognition ability of the neoepitope, but actual neoepitope immunogenicity might be influenced by many other endogenous factors, including neopeptide cleavage probability, TAP transport efficiency, peptide expression level, mutation allele fraction, and neoantigen cellular prevalence [32, 35, 36]. To this end, we proposed a quantitative score scheme, i.e., the refined immunogenicity score based on several previous studies [11, 12, 31, 37] to refine the immunogenicity of the neoepitopes identified above.

##### Refined immunogenicity score scheme

For paired peptides and MHC alleles, the following values were obtained:

*A* = Allele fraction of the mutant gene corresponding to the neoepitope

*E* = Expression level of the mutant gene, in transcript per million (TPM)

*N* = Combined score of binding affinity, proteasomal C terminal cleavage, and TAP transport efficiency, as output by *NetCTLpan* [35]

*C* = Cellular prevalence measures the percentage of tumor cells containing the identified neoantigen, as output by *PyClone* [38]

*R*_*m*_ = % percentile rank of affinity of the mutant peptide, obtained from *NetMHCpan* 4.0 [24]

*R*_*n*_ = % percentile rank of affinity of the normal peptide, obtained from *NetMHCpan* 4.0 [24]

*S* = Sequence dissimilarity between the mutant peptide and normal peptide, calculated by (1 minus sequence similarity)

*H* = T cell recognition probability of the peptide-MHC, determined by a machine-learning model using peptide hydrophobicity information

*R* = T cell recognition probability of the peptide-MHC complex, calculated by the formula 2

The refined immunogenicity score *P* was defined as:

4 $$ P=\left[A\times \tan (E)\times N\times C\right]\;\left[L\left({R}_m\right)\left(1-L\left({R}_n\right)/2\right)\times S\right]\left[H\times R\right] $$

where *L*(x) is a logistic function given by:

5 $$ L(x)=\frac{1}{1+{e}^{5\left(x-2\right)}} $$

As seen in formula (4), the refined immunogenicity score is calculated based on the product of three terms related to neoepitope processing, presentation, and recognition, including neoepitope abundance, neoepitope dissimilarity with a normal peptide, and T cell recognition probability. The first term measures the abundance of neoepitopes; here, abundance means the probability of the peptide being naturally expressed and processed before presentation by MHC-I. The expression level (*E*) of all the transcripts corresponding to the neoantigen is transformed by a hyperbolic tangent function while variant allele fraction (*A*), combined score (*N*) integratingbinding affinity, proteasomal C terminal cleavage and TAP transport efficiency, and cellular prevalence (*C*) are not manipulated. The second term is related to a potential decrease in immunogenicity of the peptide due to negative selection against cross-reacting T cells, and a sigmoidal logistic function is applied to rank the peptide-MHC binding affinity. The third term is related to the T cell recognition probability of the peptide MHC determined by the peptide hydrophobicity information and T cell cross-reacting immunogenicity, which are elaborated and calculated in the “Model building for MHC-presented and T cell-recognized neoepitope prediction” section. Finally, the immune score gives a value ranging from 0 to 1, with a higher score indicating stronger immunogenicity. The candidate neoepitope lists are then ranked by this score to obtain the final neoepitope ranking.

##### Calculation of the overall neoantigen immunogenicity score

We summed the refined immunogenicity score of all neoepitopes that were predicted to be positive in the *Pre&RecNeo* model as the so-called *the overall neoantigen immunogenicity score*. This metric measures the total immunogenicity of the neoantigen in a patient.

### Benchmarking and comparison of *pTuneos* with existing tools

A variant call format (VCF) file was generated by GATK Mutect2 as input, and *pTuneos Pre&RecNeo*, *MuPeXI*, and *Neopepsee* were run with default parameters, leading to three distinct neoantigen ranking lists. To evaluate the ranking performance of the three tools, the *RankCoverageScore* was defined as:

6 $$ {\displaystyle \begin{array}{c}\mathrm{RankCoverageScore}=\frac{\sum \limits_{n\in \mathrm{negative}}{\operatorname{rank}}_n}{T\times \mathrm{num}\;(n)}\times \mathrm{coverage}\;(n)-\frac{\sum \limits_{p\in positive}{\operatorname{rank}}_p}{T\times \mathrm{num}\;(p)}\times \mathrm{coverage}\;(p)\\ {}\mathrm{coverage}\;(k)=\frac{\max\;\left({\operatorname{rank}}_k\right)}{T}\;k\in \left(n,p\right)\end{array}} $$

where *T* denotes the total neoepitope number identified and *p* and *n* denote the set of positive and negative peptides, respectively, that were experimentally validated in vitro. The first term evaluates the rank of negative peptides considering the average percentile rank and maximum rank percentile (coverage), whereas the second term evaluates the rank of positive peptides considering the same factors. It is preferred that a positive peptide has a smaller rank value and a negative peptide has a larger rank value, indicating a better ranking result.

### Application of *pTuneos* to The Cancer Genome Atlas (TCGA) cohort study

Cohorts of patients with stomach adenocarcinoma (STAD), lung adenocarcinoma (LUAD), and skin cutaneous melanoma (SKCM), which were the most concerned cancers in cancer immunotherapy study, were obtained from The Cancer Genome Atlas (TCGA) to evaluate the association between our defined overall neoantigen immunogenicity score and several well-established immune infiltration measures including microsatellite instability status (MSI), MHC-II expression signature, and cytolytic activity (CYT). Only samples with stage III/IV characteristic were retained. Somatic mutation file in VCF format, expression profile in FPKM, and SNP 6.0 microarray data were retrieved from TCGA genomic data commons (GDC) portal. For each sample, FPKM was normalized to TPM. Segment copy number and tumor purity were estimated by ASCAT from SNP 6.0 data. Samples were excluded due to lack of accurate copy number estimation, leading to 101 LUAD samples, 166 STAD samples, and 191 SKCM samples (Additional file 4: Table S3. A, B, C). *pTuneos* was then applied to the three cohorts with mutation profile, expression profile, and copy number profile as input. For all three cohorts, MSI status of these samples was retrieved from previous study [39], as there were no samples with MSI status in SKCM and LUAD. We only applied MSI status to STAD cohort for statistic and survival analysis. For all three cohorts, immune signature associated with a 13-gene MHC II signature, which was calculated as an average gene expression of all genes in the list (Additional file 5: Table S4) [40]. Lymphocyte score was obtained from previous study [41], and we only applied this metric to SKCM cohort as the clinicopathological annotation information from frozen section slides of STAD and LUAD was not available for us to calculate the lymphocyte score. For all three cohorts, cytolytic activity (CYT) was calculated as the log-average (geometric mean) of granzyme A (*GZMA*) and perforin (*PRF1*) expression in transcripts per million (TPM) [42]. The survival data of these cohorts were also retrieved for survival analysis. We used the log-rank test and Cox proportional hazard model test to assess the correlation between all the biomarkers and overall survival (OS). The median of each metric was selected as a cutoff for high vs low separation in all biomarkers including tumor neoantigen burden (TNB), overall tumor neoantigen immunogenicity score (TNS), mutation burden, and several well-established immune infiltration measures.

### Application of *pTuneos* to immunotherapy-treated patient cohort study

Further datasets of immunotherapy-treated patients included a cohort with stage IV NSCLC treated with pembrolizumab (cohort Rizvi) [4] and two cohorts with advanced melanoma treated with anti-CTLA4 immunotherapies (cohort Snyder and cohort Van Allen) [5, 6]. In cohort Rizvi, 3 patients which did not reach 6 months’ follow-up were excluded. In cohort Snyder and cohort Van Allen, 5 patients and 7 patients were excluded due to lack of accurate copy number estimation. Final cohorts consisted of *n* = 31 Rizvi, *n* = 59 Snyder, and *n* = 103 Van Allen patients. Patient survival was the outcome measure in these cohorts. For cohort Snyder and cohort Vann Allen, overall survival (OS) was available. For cohort Rizvi, only the progression-free survival (PFS) was available. In this study, we used the log-rank test and Cox proportional hazard model test to assess the correlation between neoantigen burden and PFS or OS. We used the log-rank test and Cox proportional hazard model test to assess the correlation between the neoantigen immune score and PFS or OS. The median of each value was selected as a cutoff for high vs low separation in biomarkers including tumor neoantigen burden (TNB), overall tumor neoantigen immunogenicity score (TNS), and mutation burden. We used Wilcoxon rank sum test to determine the neoantigen burden difference between the durable clinical benefit (DCB) and no durable benefit (NDB) groups.

## Results

### General pipeline of *pTuneos*

**Fig. 1:**
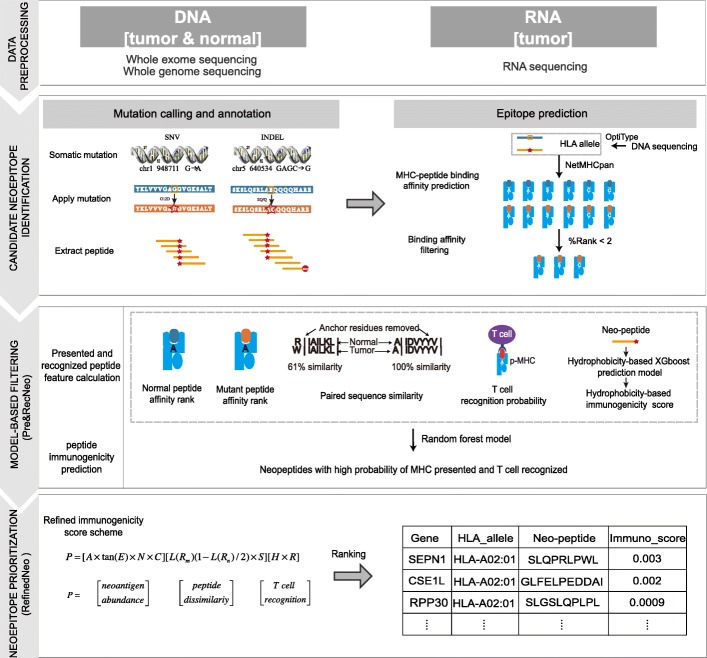
*pTuneos* comprises four steps: (1) Data preprocessing: raw sequencing data (WGS/WES and/or RNA-seq) are analyzed to identify somatic mutations (SNVs and indels) in VCF files and expression profiles. (2) Candidate neoantigen identification: for single nucleotide variants, a nucleotide change is translated into the corresponding amino acid change, which is then applied to the proteome reference, and nucleotide insertion and deletion changes are applied directly to the cDNA reference and translated into a 21-mer peptide containing the variant sites. The long peptide is then chopped up into 9–11-mer peptides. Peptide MHC binding affinities are then determined by NetMHCpan version 4.0 for both the mutant and normal peptides. (3) Model-based filtering: *pTuneos* constructs a random forest model, *Pre&RecNeo*, to predict the MHC presentation and T cell recognition probability of neopeptides based on five related features. (4) Neoepitope prioritization: *pTuneos* developed a scoring model, *RefinedNeo*, to refine the rank the of neoepitope immunogenicity, which represents the probability of naturally processed, MHC-presented, and T cell-recognized neopeptide and the actual immunologic effects of a neopeptide in clinical tumor treatment

The *pTuneos* workflow consists of four steps (Fig. 1): data preprocessing, candidate neoepitope identification, model-based filtering, and neoepitope prioritization based on the refined immunogenicity score.

In the first step, raw sequencing data (WGS/WES and/or RNA-seq) are analyzed to identify somatic mutations (SNVs and indels) in a VCF file and expression profile. HLA alleles are determined from WGS/WES and/or RNA-seq data by OptiType. Second, for SNVs, the nucleotide change is translated into the corresponding amino acid change, which is then applied to the proteome reference, and nucleotide insertion and deletion changes are applied directly to the cDNA reference and translated into a 21-mer peptide containing variant sites. The long peptide is then chopped up into 9–11-mer long peptides. Peptide-MHC binding affinities for both mutant and normal peptides are then determined by NetMHCpan version 4.0. In addition, *pTuneos* adopts several preliminary filtering strategies to obtain reliable neo-epitopes: (1) sequence coverage and gene variant allele frequency, (2) %rank affinity of mutant peptides, and (3) gene expression level of corresponding mutant peptides. Third, *pTuneos* constructs a random forest model, *Pre&RecNeo*, to predict the MHC presentation and T cell recognition probability of neopeptides based on five related features. Finally, based on the candidate neoepitopes identified in the third step, we developed an efficient scoring model, *RefinedNeo*, which calculates a refined immunogenicity score reflecting the probability of naturally processed, MHC-presented, and T cell-recognized neopeptide and the actual immunologic effects of a neopeptide in clinical tumor treatment. Efficient computational techniques were incorporated in *pTuneos* including (1) the training of state-of-the-art machine learning model for MHC-presented and T cell-recognized neoepitope immunogenicity prediction; (2) handling data imbalance issue in model building; (3) defining a novel score scheme for naturally processed, presented, and T cell-recognized neoepitope immunogenicity prioritizing; and (4) defining a quantitative measurement for neoepitope prioritization performance evaluation. Detailed information can be referred in the “Implementation” section.

### Neoepitope prioritization performance evaluation of *pTuneos* in melanoma dataset

To evaluate the neoepitope prioritization performance of *pTuneos*, we applied it to a public dataset containing three samples from a recent study that reported experimentally confirmed immunogenic and non-immunogenic peptides in melanoma [32]. The MHC-Dextramer assay confirmed that 7 of 21 peptides can induce T cell recognition, whereas the tandem mini-gene constructs (TMC) transfection assay confirmed that only 5 of 7 immunogenic peptides could be naturally processed, presented, and recognized (Additional file 3: Table S2).

**Fig. 2:**
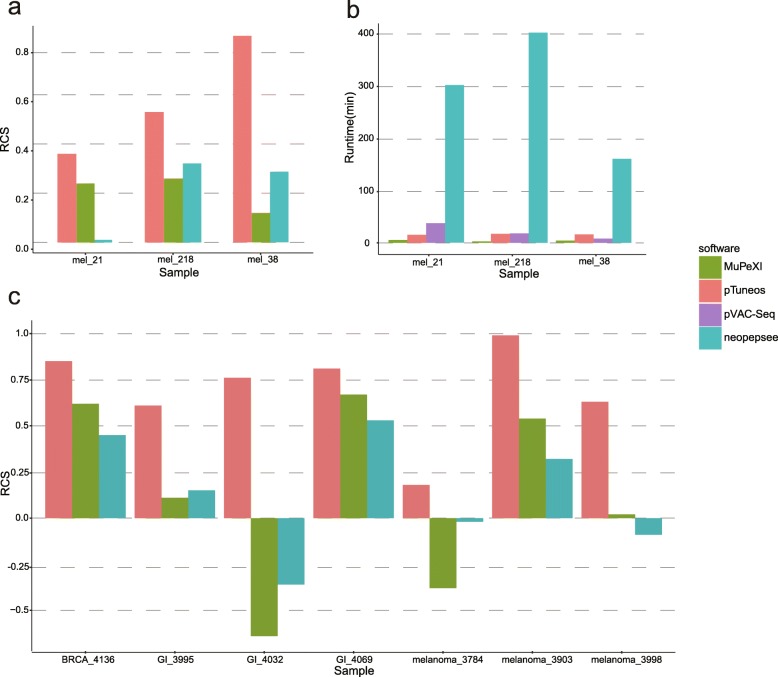
**a** *Rank coverage score (RCS)* of the final rank list obtained from *pTuneos Pre&RecNeo*, *MuPeXI*, and *Neopepsee*, tested on the MEL dataset. **b** Runtime efficiency comparison of *pTuneos*, *MuPeXI, pVAC-Seq*, *and Neopepsee* tested on the MEL dataset. **c** *Rank coverage score (RCS)* of the final rank list obtained from *pTuneos MuPeXI*, and *Neopepsee*, tested on the TILs stimulation datasets

Firstly, the *pTuneos Pre&RecNeo* module, *MuPeXI*, and *Neopepsee* were all run on this dataset and led to different numbers of candidate neopeptides (Additional file 6: Table S5. A-I). We then defined a *RankCoverageScore* (see the “Implementation” section) to comprehensively and objectively evaluate the rank results of the final neopeptide list derived from the three tools. Among them, *pTuneos Pre&RecNeo* obtained a higher *RankCoverageScore* than either *MuPeXI* or *Neopepsee* (Fig. 2a), indicating that *pTuneos Pre&RecNeo* could identify MHC-presented and T cell-recognized neopeptides more effectively than existing in silico tools.

Secondly*,* the *pTuneos RefinedNeo* module was applied to the candidate neopeptides obtained from *pTuneos Pre&RecNeo* to evaluate their immunogenicity considering neopeptide naturally processed and presented, leading to a refined rank of candidate neoepitopes for in vivo clinical application. In the original study, the neopeptides OR8B3_T109I (identified from sample MEL_38) and MRPS5_P59L (identified from sample MEL_218) were not processed and presented from endogenously expressed proteins. In our study, *pTuneos RefinedNeo* successfully refined the rank of OR8B3_T109I from 16 to 31 (total 40) and the rank of MRPS5_P59L from 3 to 58 (total 70) (Additional file 7: Table S6. A, B), demonstrating that *pTuneos RefinedNeo* could help to further filter out those neopeptides not naturally processed and presented in cancer immunotherapy.

### Runtime benchmark of *pTuneos* with existing tools

Runtime is an important issue for successful clinical application of neoantigen-based vaccine design. As *pTuneos* implemented multi-process programming, it completes the whole candidate neoepitope identification and prioritization procedure in a short time. In this study, we benchmark the runtime efficiency of *pTuneos* with existing in silico neoantigen identification tools starting with the list of variants identified by *Mutect2* and the expression profile identified by *Kallisto* from three melanoma patients (phs001005.v1.p1) (Fig. 2b). The benchmark results indicated that *pTuneos* is comparable to *MuPeXI* and *pVAC-Seq* and is much faster than *Neopepsee*. Specifically, *pTuneos* was 20 times faster than *Neopepsee*, both taken as the model-based tools.

### Performance evaluation of *pTuneos* using naturally processed and presented neopeptides

We continue to evaluate the performance of *pTuneos* in 3 public datasets containing 7 samples from recent studies that reported 16 MHC-I naturally processed and presented neopeptides recognized by tumor-infiltrating lymphocytes (TILs), which are obtained from tandem mini-gene constructs (TMC) transfection assays (Additional file 8: Table S7) [43–45]. *pTuneos*, *MuPeXI*, and *Neopepsee* were all run on this dataset respectively and led to different numbers of candidate neopeptides (Additional file 9: Table S8. A, B, C). Here, we obtained the final rank of neopeptides firstly by *Pre&RecNeo* module and refined by the *RefinedNeo* module. We still utilized the *RankCoverageScore* (see the “Implementation” section) to evaluate the rank results of the final neopeptide list derived from these three tools. Among them, *pTuneos* obtained a higher *RankCoverageScore* than either *MuPeXI* or *Neopepsee* (Fig. 2c), indicating that *pTuneos* could identify naturally processed, presented, and TIL-recognized neopeptides more effectively than existing in silico tools.

### Application of *pTuneos* to TCGA cohort study

We first applied *pTuneos* to TCGA cancer cohorts with stage III/IV stomach adenocarcinoma (STAD; *n* = 166/441), stage III/IV lung adenocarcinoma (LUAD; *n* = 101/569), and stage III/IV cutaneous melanoma (SKCM; *n* = 191/470) (Additional file 4: Table S3. A, B, C) to explore the relationship among the overall neoantigen immunogenicity score (defined by the sum of the refined immunogenicity score of all identified neopeptides, see the “Implementation” section), the identified neoantigen burden, microsatellite instability status, and several immune infiltration measurements. The overall survival (OS) prediction powers of these measurements were also compared.

**Fig. 3:**
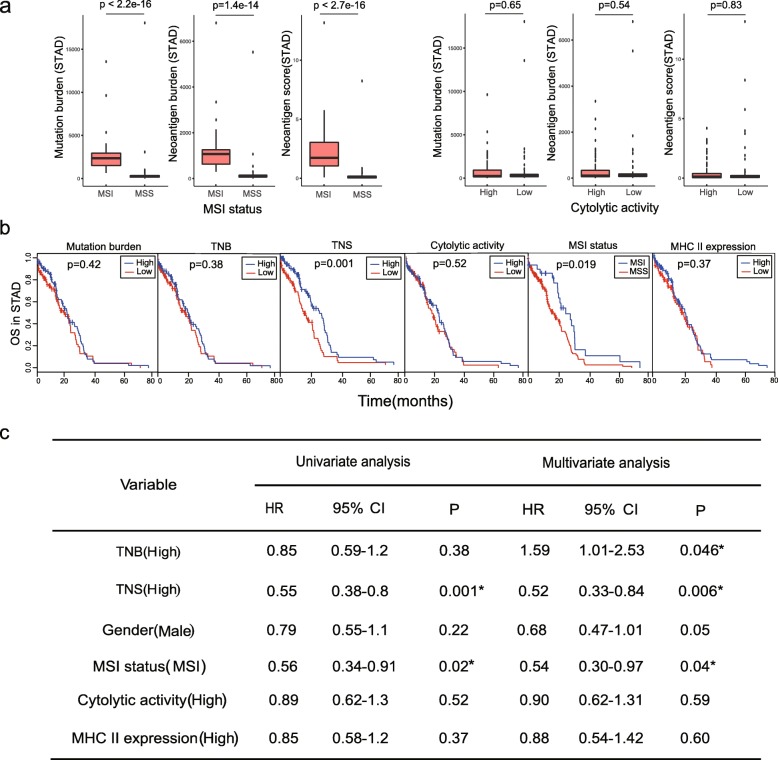
**a** MSI status showed strong correlation with neoantigen burden and overall neoantigen immunogenicity score while cytolytic activity did not in stomach adenocarcinoma. **b** Kaplan-Meier estimates of overall survival according to mutation burden, tumor neoantigen burden (TNB), overall tumor neoantigen immunogenicity score (TNS), cytolytic activity, MSI status, and MHC II expression. The median of each value was selected as a cutoff for high vs low separation in all biomarkers except MSI status classified as MSI and MSS groups. **c** Univariate and multivariate Cox regression survival analyses of TCGA STAD data on different single biomarkers and all. TNB, tumor neoantigen burden; TNS, overall tumor neoantigen immunogenicity score; MSI, microsatellite instability; MSS, microsatellite stability; HR, hazard ratio; CI, confidence interval

In STAD, several well-established markers have been reported to correlate with the clinical outcome of immunotherapy. For example, MSI status has been shown to correlate with a better clinical outcome [39]. Cytolytic activity (CYT) defined as the transcript levels of two key cytolytic effectors, i.e., granzyme A (GZMA) and perforin (PRF1), was also reported to be an indicator of CD8+ T cell activation [42]. In our study, MSI status was found to show a strong correlation with our identified neoantigen burden and the overall neoantigen immunogenicity score calculated by *pTuneos*, while cytolytic activity did not (Fig. 3a). Furthermore, high overall neoantigen immunogenicity score (> median) was associated with the overall survival by both the univariate and multivariate Cox regression analyses, while well-established markers including cytolytic activity and MHC II expression were not significantly associated with overall survival (Fig. 3b, c), indicating that our defined overall neoantigen immunogenicity score is a reliable predictive biomarker in STAD survival analysis.

**Fig. 4:**
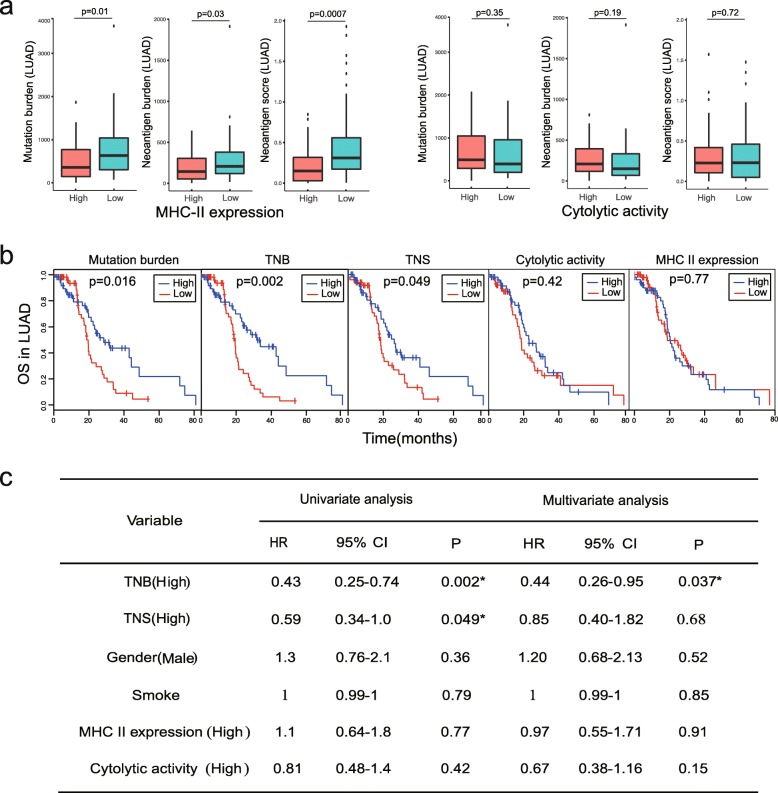
**a** High MHC-II expression score (> median) is more significantly correlated with low overall neoantigen immunogenicity score (*p* = 0.0007) than mutation burden and neoantigen burden, while cytolytic activity did not correlate with mutation burden, neoantigen burden, or overall neoantigen immunogenicity score in lung adenocarcinoma. **b** Kaplan-Meier estimates of overall survival according to mutation burden, tumor neoantigen burden (TNB), overall tumor neoantigen immunogenicity score (TNS), cytolytic activity, and MHC-II expression. The median of each value was selected as the cutoff for high vs low separation in all biomarkers. **c** Univariate and multivariate Cox regression survival analyses of TCGA LUAD data on different single biomarkers and all. TNB, tumor neoantigen burden; TNS, overall tumor neoantigen immunogenicity score; HR, hazard ratio; CI, confidence interval

In LUAD, a 13 gene MHC-II expression signature was also reported to correlate with immune infiltration. This signature was presented to be a marker of immune activity [40]. Based on TCGA RNA-sequencing data, we explored the relationship between MHC-II expression signature, the cytolytic activity, and the overall neoantigen immunogenicity score. Our study indicated that high MHC-II expression score (> median) is more significantly correlated with low overall neoantigen immunogenicity score (*p* = 0.0007) than mutation burden and neoantigen burden. Having stratified the cohort by cytolytic activity score, cytolytic activity did not correlate with mutation burden, neoantigen burden, or overall neoantigen immunogenicity score (Fig. 4a). We found that mutation burden, neoantigen burden, and the overall neoantigen immunogenicity score (> median) all exhibited certain prognosis ability while cytolytic activity and MHC II expression were not significantly associated with overall survival (Fig. 4b). In the Cox regression analysis, the tumor neoantigen burden and the overall neoantigen immunogenicity score calculated by *pTuneos* were all identified as two independent prognostic factors for overall survival analysis (Fig. 4c).

**Fig. 5:**
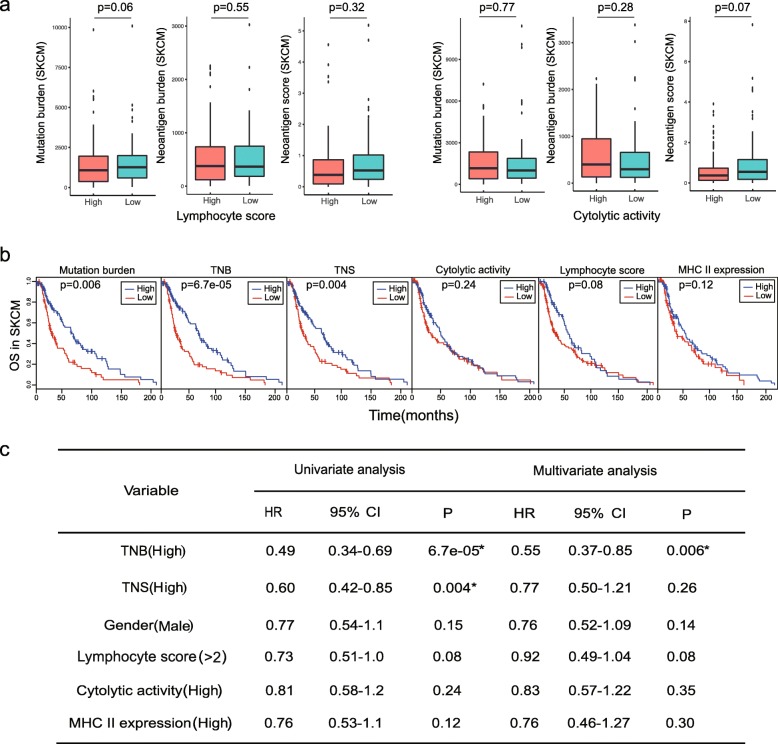
**a** Neither lymphocyte nor cytolytic activity showed correlation with neoantigen burden or overall neoantigen immunogenicity score in cutaneous melanoma. **b** Kaplan-Meier estimates of overall survival according to mutation burden, tumor neoantigen burden (TNB), overall tumor neoantigen immunogenicity score (TNS), cytolytic activity, lymphocyte score, and MHC II expression. The median of each value was selected as a cutoff for high vs low separation in all biomarkers. **c** Univariate and multivariate Cox regression survival analyses of TCGA SKCM data on different single biomarkers and all. TNB, tumor neoantigen burden; TNS, overall tumor neoantigen immunogenicity score; HR, hazard ratio; CI, confidence interval

In SKCM, lymphocyte density and distribution were previously measured to define a semi-quantitative lymphocyte score representing lymphocyte infiltration [41]. In our study, neither lymphocyte nor cytolytic activity showed correlation with neoantigen burden or overall neoantigen immunogenicity score (Fig. 5a). Nevertheless, high neoantigen burden (> median) exhibited improved overall survival in both univariate and multivariate analyses, and the high overall neoantigen immunogenicity score (> median) was associated with overall survival in univariate analysis, while well-established markers including cytolytic activity and MHC II expression were not significantly associated with overall survival (Fig. 5b, c).

Taking together, these results showed that traditional well-established markers exhibited limitations in survival prediction among different cancer types, whereas only the overall neoantigen immunogenicity score calculated by *pTuneos* could be predictive of survival in all three TCGA cohorts, indicating the potential power of the overall neoantigen immunogenicity score as a pan-cancer predictive biomarker in cancer survival analysis.

### Application of *pTuneos* to the immunotherapy-treated patient cohorts study

To further assess the effectiveness and robustness of *pTuneos*, we also applied the whole pipeline to three independent datasets comprising anti-CTLA-4-treated melanoma patients and anti-PD-1-treated lung cancer patients to compare the identified neoantigen profile with patient survival patterns [4–6].

**Fig. 6:**
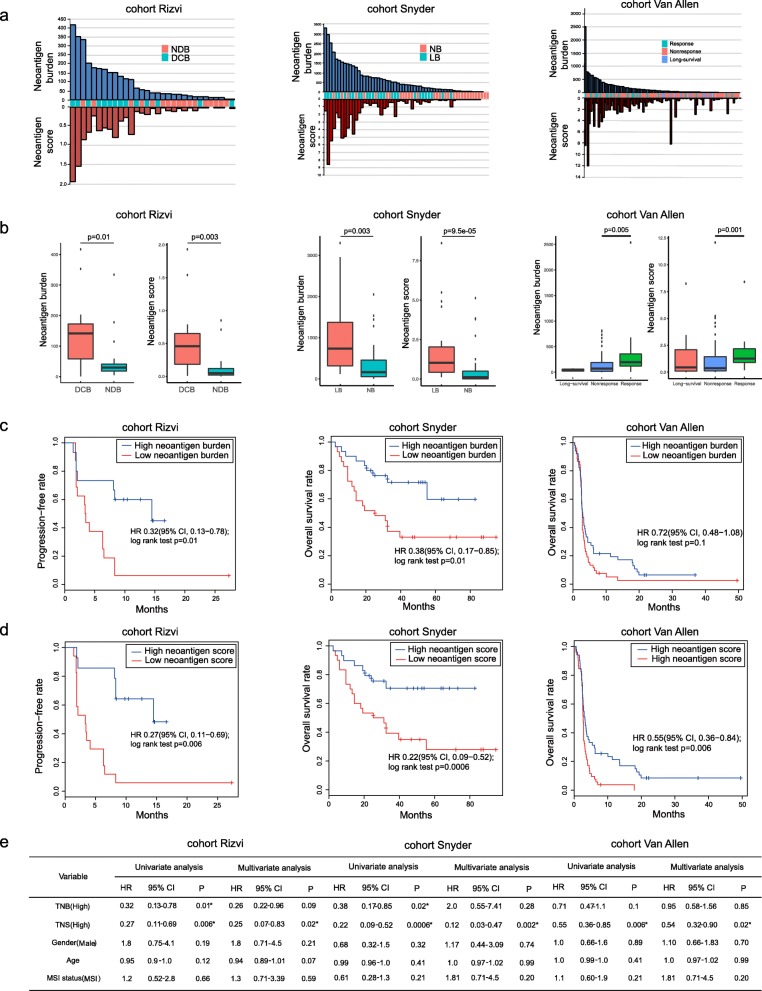
**a** Neoantigen burden and neoantigen immunogenicity score distribution in three immunotherapy-treated patient cohorts. **b** The overall neoantigen immunogenicity score showed more difference than neoantigen burden between long benefit group and no benefit group. **c** In cohort Rizvi and Snyder, a high neoantigen burden (> median) was associated with improved progression-free survival (PFS) or overall survival (OS) (cohort Rizvi: hazard ratio [HR] 0.32, 95% confidence interval [CI] 0.13 to 0.78, log-rank *P* = 0.01; cohort Snyder: HR 0.38, 95% CI 0.17 to 0.85, log-rank *P* = 0.01) whereas in cohort Van Allen, neoantigen burden was not associated with improved OS (HR 0.72, 95% CI 0.48 to 1.08, log-rank *P* = 0.1). **d** High overall neoantigen immunogenicity score (> median) shows more significant patient PFS or OS separations than those based purely on neoantigen burden (cohort Rizvi: HR 0.27 [95% CI, 0.11–0.69], log-rank *P* = 0.006; cohort Snyder: HR 0.22 [95% CI, 0.09–0.52], log-rank *P* = 0.0006; cohort Van Allen: HR 0.55 [95% CI, 0.36–0.84], log-rank *P* = 0.006). **e** Univariate Cox regression and multivariate Cox regression analyses showed that only overall neoantigen immunogenicity score was associated with improved PFS or OS. NDB, no durable benefit; DCB, durable clinical benefit; NB, no benefit; LB, long benefit; PFS, progression-free survival; OS, overall survival; TNB, tumor neoantigen burden; TNS, overall tumor neoantigen immunogenicity score; HR, hazard ratio; CI, confidence interval

We first explored the difference between tumor neoantigen burden profile and the overall neoantigen immunogenicity score profile (see the “Implementation” section). For the cohort of 31 patients with lung cancer treated with pembrolizumab (cohort Rizvi), 14 patients had a durable clinical benefit (DCB) and 17 patients had no durable benefit (NDB). *pTuneos* identified a median of 41 candidate neoantigens per tumor (range 1–417), and the overall neoantigen immunogenicity score ranges from 0 to 1.93 (Additional file 10: Table S9. A; Fig. 6a). For a cohort of 59 patients with melanoma treated with ipilimumab or tremelimumab (cohort Snyder), 36 patients had a DCB and 23 patients had NDB. *pTuneos* identified a median of 384 candidate neoantigens per tumor (range 0–3299), and the overall neoantigen immunogenicity score ranges from 0 to 8.6 (Additional file 10: Table S9, B; Fig. 6a). For the cohort of 103 patients with melanoma treated by ipilimumab (cohort Van Allen), 21 patients had a DCB and 72 patients had NDB. *pTuneos* identified a median of 74 candidate neoantigens per tumor (range 0–2537) and the overall neoantigen immunogenicity score ranges from 0 to 12.07 (Additional file 10: Table S9, C; Fig. 6a). In all three cohorts, the neoantigen immunogenicity score showed more difference than neoantigen burden between long benefit group and no benefit group (Fig. 6b).

Next, we evaluated the different survival prediction power of the neoantigen burden, the overall neoantigen immunogenicity score, and the MSI status. For cohort Rizvi and Snyder, a high neoantigen burden (> median) was associated with improved progression-free survival (PFS) or overall survival (OS) (cohort Rizvi: hazard ratio [HR] 0.32, 95% confidence interval [CI] 0.13 to 0.78, log-rank *P* = 0.01; cohort Snyder: HR 0.38, 95% CI 0.17 to 0.85, log-rank *P* = 0.01), whereas in cohort Van Allen, neoantigen burden was not associated with improved overall survival (OS) (HR 0.72, 95% CI 0.48 to 1.08, log-rank *P* = 0.1) (Fig. 6c). Based on the above-identified neoantigens, the overall neoantigen immunogenicity score was calculated across the three cohorts of patients. In all three cohorts, the overall neoantigen immunogenicity score shows more significant patient progression-free survival (PFS) or overall survival (OS) separations than those based purely on neoantigen burden (cohort Rizvi: HR 0.27 [95% CI, 0.11–0.69], log-rank *P* = 0.006; cohort Snyder: HR 0.22 [95% CI, 0.09–0.52], log-rank *P* = 0.0006; cohort Van Allen: HR 0.55 [95% CI, 0.36–0.84], log-rank *P* = 0.006; Fig. 6d). The MSI status was also calculated across three cohorts. Univariate Cox regression and multivariate Cox regression analyses showed that only overall neoantigen immunogenicity score was associated with improved progression-free survival (PFS) or overall survival (OS) (Fig. 6e). These results not only validated the rationality of the definition of the refined neoantigen immunogenicity score, but also demonstrated that the overall neoantigen immunogenicity score could be predictive of survival after checkpoint blockade immunotherapy.

We finally checked the 3 neoepitopes which were validated using multimer or restimulation assays in the cohort Rizvi and cohort Snyder in the identified neoantigen list obtained from *pTuneos*. In the cohort Rizvi, the neopeptide ASNA**S**SAAK derived from *HERC1* (p.P3278S) mutation in patient CA9903 was revealed to elicit T cell response using multimer assays. In the identified neoantigen list obtained from *pTuneos Pre&RecNeo* module, this neopeptide ranked in the first place (Additional file 11: Table S10. A). In the cohort Snyder, there are two neopeptides which were found to have polyfunctional T cell responses using intracellular cytokine staining (ICS) assay. The first neopeptide T**E**SPEEQHI results from *FAM3C* (p.K193E) mutation in patient CR9306. In the identified neoantigen list obtained from *pTuneos Pre&RecNeo* module, this neopeptide ranked in the second place (Additional file 11: Table S10. B). The second neopeptide GLER**E**GFTF results from *CSMD1* (p.G3446E) mutation in patient CR0095. However, we could not find this peptide in the final list from *pTuneos Pre&RecNeo* module (Additional file 11: Table S10. C). We found that the predicted MHC class I binding affinity %rank between GLER**E**GFTF and MHC-I alleles (A0201, A3101, B3502, B3906, C0401, C0702) was all greater than 2 predicted by NetMHCpan 4.0, which means that it could not be presented by MHC-I molecules and it was filtered by *pTuneos* in the epitope identification step. Taking together, *pTuneos* could identify 2 out of 3 validated neoantigen and rank them at the top of final list, demonstrating its effectiveness. Notably, in the original study [5], researchers also found that all the predicted MHC class I affinity of this neopeptide are greater than 500 nM by NetMHC 3.4. These results indicated that the sensitivity of peptide-MHC-I binding affinity prediction methods such as NetMHC and NetMHCpan is needed to be improved, and the low predicted binding affinity of peptide-MHC-I does not necessarily indicate that they could not activate T cell response.

## Discussion

In the neoantigen profile analysis of TCGA cohorts, the overall tumor neoantigen immunogenicity score (TNS) was demonstrated to be an efficient survival predictive biomarker in all three cancer types through univariate analysis. However, in LUAD, TNS barely reached significance in univariate analysis (*p* = 0.049; Fig. 4b, c) and did not reach a significance in multivariate analysis while tumor neoantigen burden (TNB) was significantly associated with overall survival. Similarly, in SKCM, TNS did not achieve a significance in multivariate analysis and underperformed in univariate analysis compared with tumor neoantigen burden (TNB) (Fig. 5b, c). These findings suggested that although TNS is predictive of survival for TCGA patient cohorts, TNB is a better biomarker of overall survival in LUAD and SKCM.

It is anticipated that MSI correlated with neoantigen burden and overall neoantigen immunogenicity in STAD as MSI status contributes to the generation of gene mutation and leads to production of more potential neoantigens, while cytolytic activity, which reflects the activity of immune infiltrate T cells, did not exhibit this correlation. This could be explained by the model for evolution of tumor-immune associations proposed by Rooney et al. [42]. In the early stage of tumor development, intrinsic tumor factors such as neoantigens or viruses induce local immune infiltrates. These factors are expected to be correlated with CYT. However, with the development of tumor and the accumulation of resistance mutation such as p53, ALOX, and IDO1, these mutations would suppress the immune infiltrate, leading to a low CYT or even showing no correlation between neoantigens burden and CYT. In our analysis, we only selected those samples with stage III/IV characteristic, and these samples are at late stage of tumor progression. According to the model proposed by Rooney et al., it is reasonable that neoantigen burden and overall neoantigen immunogenicity did not correlate with cytolytic activity in our study. Kim et al. [14] also found that cytolytic activity was not associated with survival prognosis in STAD.

Future development of *pTuneos* will include four main aspects: (1) Currently, *pTuneos* predicts the presentation and recognition probability of neopeptide utilizing machine learning model based on peptides retrieved from MHC multimer assays and defined a refined immunogenicity score to evaluate the immunogenicity of the naturally processed and presented neoantigens. As more and more datasets are available containing confirmed immunogenic peptides that are naturally processed, presented, and TIL-recognized utilizing tandem mini-gene constructs (TMC) transfection assay [46], the future updates of *pTuneos* is to build a learning model to identify such neopeptides; (2) investigation of MHC-II binding peptide identification and evaluation [47–49]; (3) incorporation of mass spectroscopy data processing into the pipeline for further filtering of neoantigen candidates; and (4) identification of other types of neoantigens besides SNV-based neoantigens [55], like gene fusion-based [50], RNA alternative splicing-based [51], and RNA editing-based neoantigens [52, 53].

## Conclusions

In summary, *pTuneos* was demonstrated to be a state-of-the-art one-stop in silico prediction tool for identifying and prioritizing cancer neoantigens compared with other available tools in terms of neoantigen prioritization performance and runtime efficiency. Based on the putative neoantigens obtained by high-peptide-MHC binding affinity, *pTuneos* implemented a two-step filtering and ranking strategy to prioritize neoantigens. In addition, the *pTuneos Pre&RecNeo* module could eliminate neoantigens which were not presented and T cell-recognized, and the *pTuneos RefinedNeo* module could refine the ranking list by prioritizing the actual neoantigen immunogenicity. We validated the reasonability of this strategy by applying it to an independent dataset containing three samples from a recent study with experimentally confirmed immunogenic and non-immunogenic peptides in melanoma. We also validated its ability by evaluating the immunogenicity of naturally processed and presented neoantigens with TIL recognitions from three additional datasets. We further demonstrated the utility of *pTuneos* by applying it to TCGA cohorts and three cohorts undergoing checkpoint blockade immunotherapy and revealed that the overall neoantigen immunogenicity score was more predictive of patient survival than the neoantigen burden and other well-established markers. Taking together, *pTuneos* will enable the efficient identification and prioritization of personal neoantigens for improved personalized vaccine design in cancer immunotherapy.

## Availability and requirements

Project name: pTuneos

Project home page: https://github.com/bm2-lab/pTuneos

Operating system(s): Linux

Programming language: python 2.7, R 3.4

Other requirements: Java 1.8 or higher

License: GNU license - GPL 2.0 (GNU General Public License. version 2) (https://opensource.org/licenses/GPL-2.0)

Any restrictions to use by non-academics: none

## Supplementary information

**Additional file 1:** **Figure S1.** A. Pipeline of calculating hydrophobicity immunogenicity score. B-D. Performance of three models corresponding to 9mer, 10 mer and 11mer peptides.

**Additional file 2:** **Table S1.** Descriptions of training data used in *pTuneos Pre&RecNeo*.

**Additional file 3:** **Table S2.** Experimentally confirmed immunogenic and non-immunogenic peptides in melanoma from *Carreno* et al. (ref. [15]).

**Additional file 4:** **Table S3.** A. Clinical information and neoantigen information of stage III/IV stomach adenocarcinoma (*n*=166). B. Clinical information and neoantigen information of stage III/IV lung adenocarcinoma (*n*=101). C. Clinical information and neoantigen information of stage III/IV cutaneous melanoma (*n*=191).

**Additional file 5:** **Table S4.** A list of 13-gene MHC II signature which associated with immune signature.

**Additional file 6:** **Table S5.** A. Candidate neoepitopes identified using *pTuneos Pre&RecNeo* from MEL_21. B. Candidate neoepitopes identified using *pTuneos Pre&RecNeo* from MEL_38. C. Candidate neoepitopes identified using *pTuneos Pre&RecNeo* from MEL_218. D. Candidate neoepitopes identified using *MuPeXI* from MEL_21 (only retain neoantigen priority score>0). E. Candidate neoepitopes identified using *MuPeXI* from MEL_38 (only retain neoantigen priority score>0). F. Candidate neoepitopes identified using *MuPeXI* from MEL_218 (only retain neoantigen priority score>0). G. Candidate neoepitopes identified using *neopepsee* from MEL_21 (only retain neoantigen predition level is high or medium). H. Candidate neoepitopes identified using *neopepsee* from MEL_38 (only retain neoantigen predition level is high or medium). I. Candidate neoepitopes identified using *neopepsee* from MEL_218 (only retain neoantigen predition level is high or medium).

**Additional file 7:** **Table S6.** A. Refined neoepitope rank obtained by *pTuneos RefinedNeo* of MEL_38. B. Refined neoepitope rank obtained by *pTuneos RefinedNeo* of MEL_218.

**Additional file 8:** **Table S7.** Experimentally confirmed immunogenic and non-immunogenic peptides in Zacharakis et al., Tran et al. and Gros et al.

**Additional file 9:** **Table S8.** A. Candidate neoepitopes identified using *pTuneos* from 7 samples in Zacharakis et al., Tran et al. and Gros et al. B. Candidate neoepitopes identified using *MuPeXI* from 7 samples in Zacharakis et al., Tran et al. and Gros et al. (only retain neoantigen priority score>0). C. Candidate neoepitopes identified using *neopepsee* from 7 samples in Zacharakis et al., Tran et al. and Gros et al. (only retain neoantigen predition level is high or medium).

**Additional file 10:** **Table S9.** A. Clinical information and neoantigen information of cohort Rizvi (*n*=31). B. Clinical information and neoantigen information of cohort Snyder (*n*=59). C. Clinical information and neoantigen information of cohort Van Allen (*n*=103).

**Additional file 11:** **Table S10.** A. Candidate neoepitopes information obtained from patient CA9903 in Rizvi cohort using *pTuneos Pre&RecNeo*. B. Candidate neoepitopes information obtained from patient CR9306 in Snyder cohort using *pTuneos Pre&RecNeo*. C. Candidate neoepitopes information obtained from patient CR0095 in Snyder cohort using *pTuneos Pre&RecNeo*.

## Data Availability

Sequencing data for model validation was retrieved from dbGap (accession number phs001005.v1.p1). Sequencing data for 3 datasets of naturally processed and presented neopeptides recognized by TILs were retrieved from National Center for Biotechnology Information Bioproject database (accession number PRJNA298310 [43]; accession number PRJNA298330 [44]; accession number PRJNA342632 for exome data and accession number PRJNA243084 for RNA-seq data [45]). Sequencing data from the three cohorts are also obtained from dbGap (accession number phs000980.v1.p1 [4]; accession number phs001041.v1.p1 [5]; accession number phs000452.v2.p1 [6]). The survival data for these cohorts were also retrieved from the three studies.

## Abbreviations

AUC: Area under the curve
BA: Binding affinity
CYT: Cytolytic activity
DCB: Durable clinical benefit
FPKM: Fragments per kilobase million
GDC: Genomic data commons
GZMA: Granzyme A
HLA: Human leukocyte antigen
HR: Hazard ratio
ICS: Intracellular cytokine staining
IEDB: Immune Epitope Database
LUAD: Lung adenocarcinoma
MHC: Major histocompatibility complex
MSI: Microsatellite instability
NDB: No durable benefit
NSCLC: Non-small cell lung cancer
OS: Overall survival
PFS: Progression-free survival
PRF1: Perforin 1
RF: Random forest
SKCM: Skin cutaneous melanoma
SMOTE: Synthetic Minority Over-sampling Technique
SNV: Single-nucleotide variant
STAD: Stomach adenocarcinoma
TAP: Transporter associated with antigen processing
TCGA: The Cancer Genome Atlas
TCR: T cell receptor
TIL: Tumor-infiltrating lymphocyte
TMC: Tandem mini-gene constructs
TNB: Tumor neoantigen burden
TNS: Overall tumor neoantigen immunogenicity score
TPM: Transcript per million
VCF: Variant call format
VEP: Variant Effect Predictor
WGS/WES: Whole-genome or whole-exome sequencing
XGBoost: eXtreme Gradient Boosting
