# Supplementary figures and images for: pTuneos: prioritizing tumor neoantigens from next-generation sequencing data
Source: Genome Med. 2019 Oct 30;11:67. doi: 10.1186/s13073-019-0679-x (PMC6822339; doi:10.1186/s13073-019-0679-x)

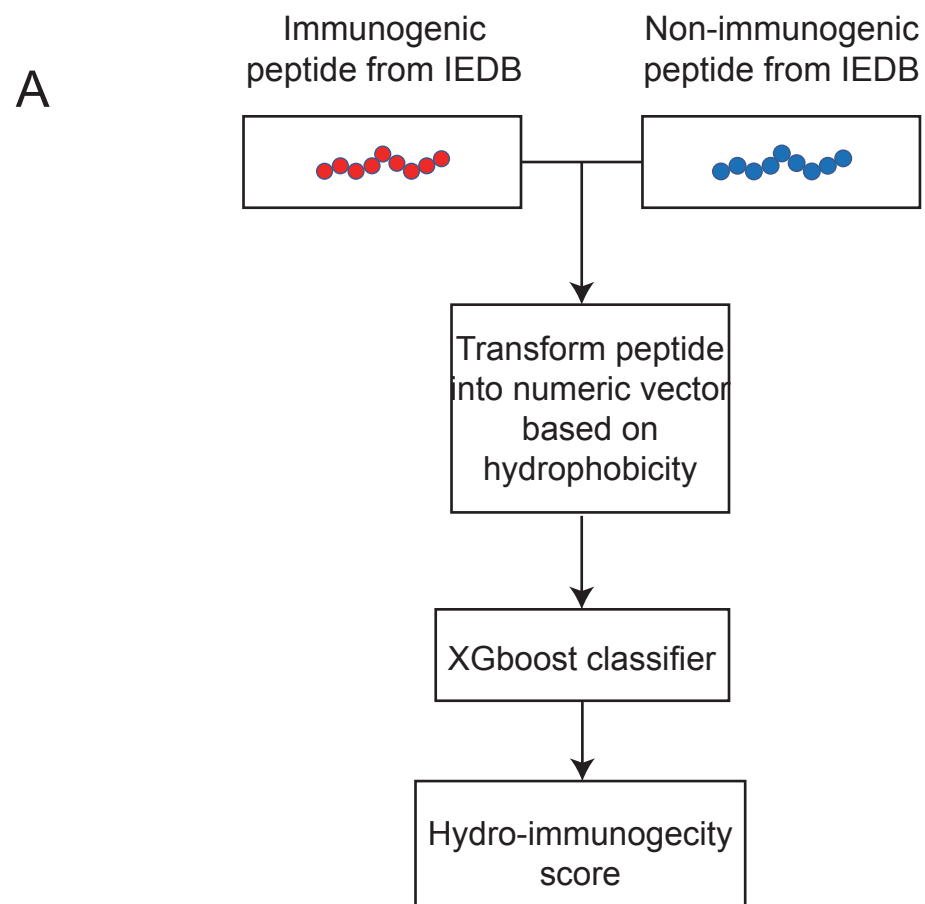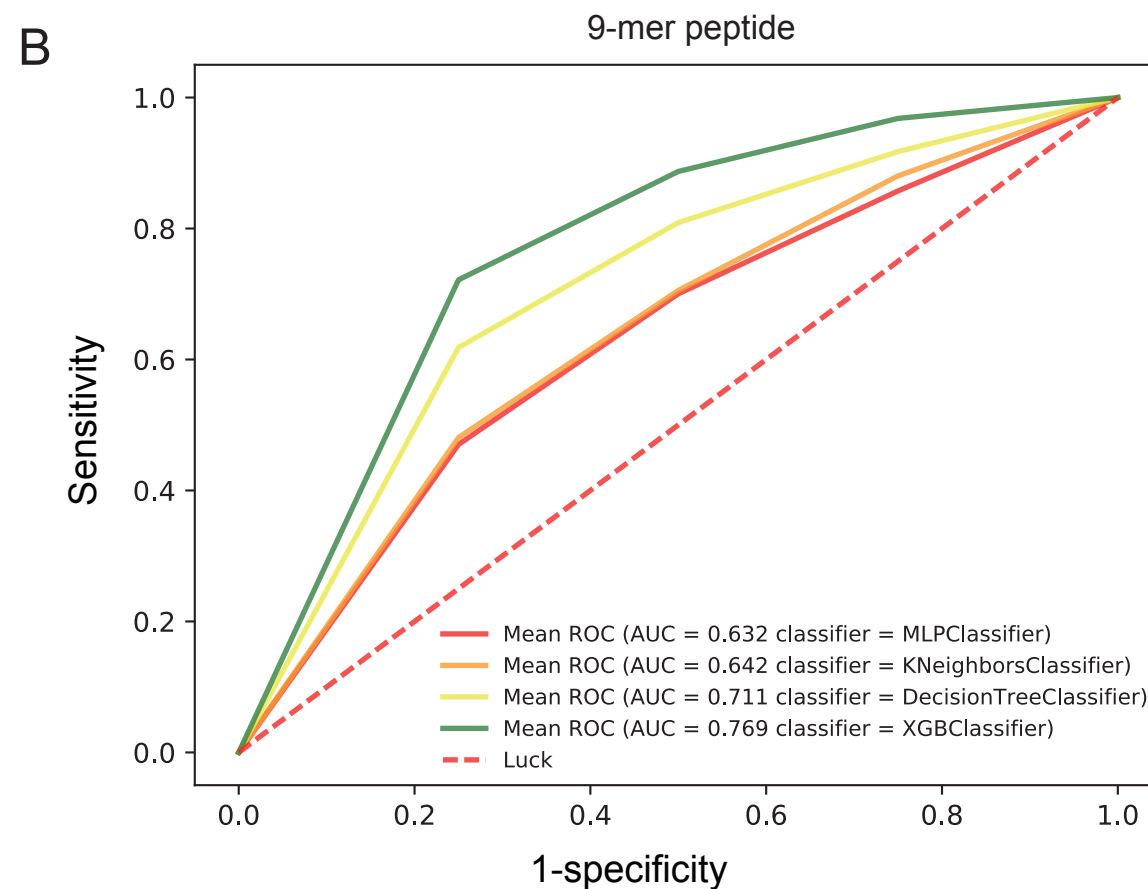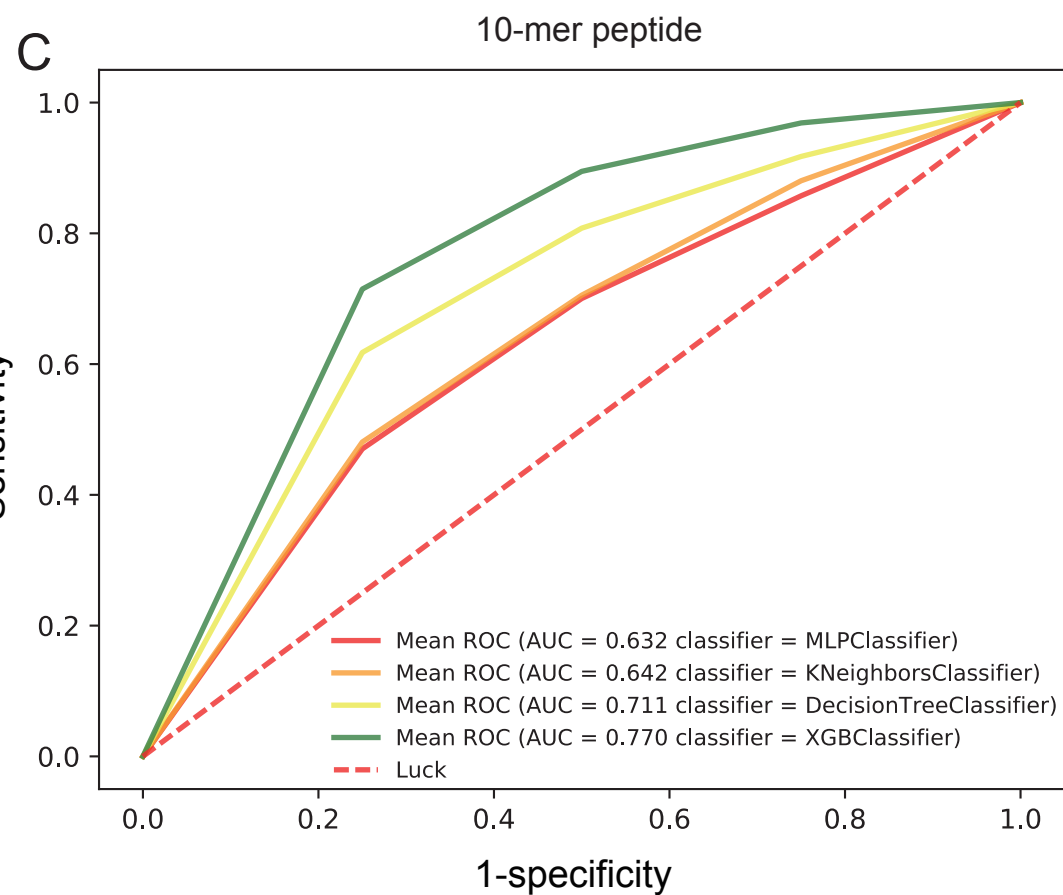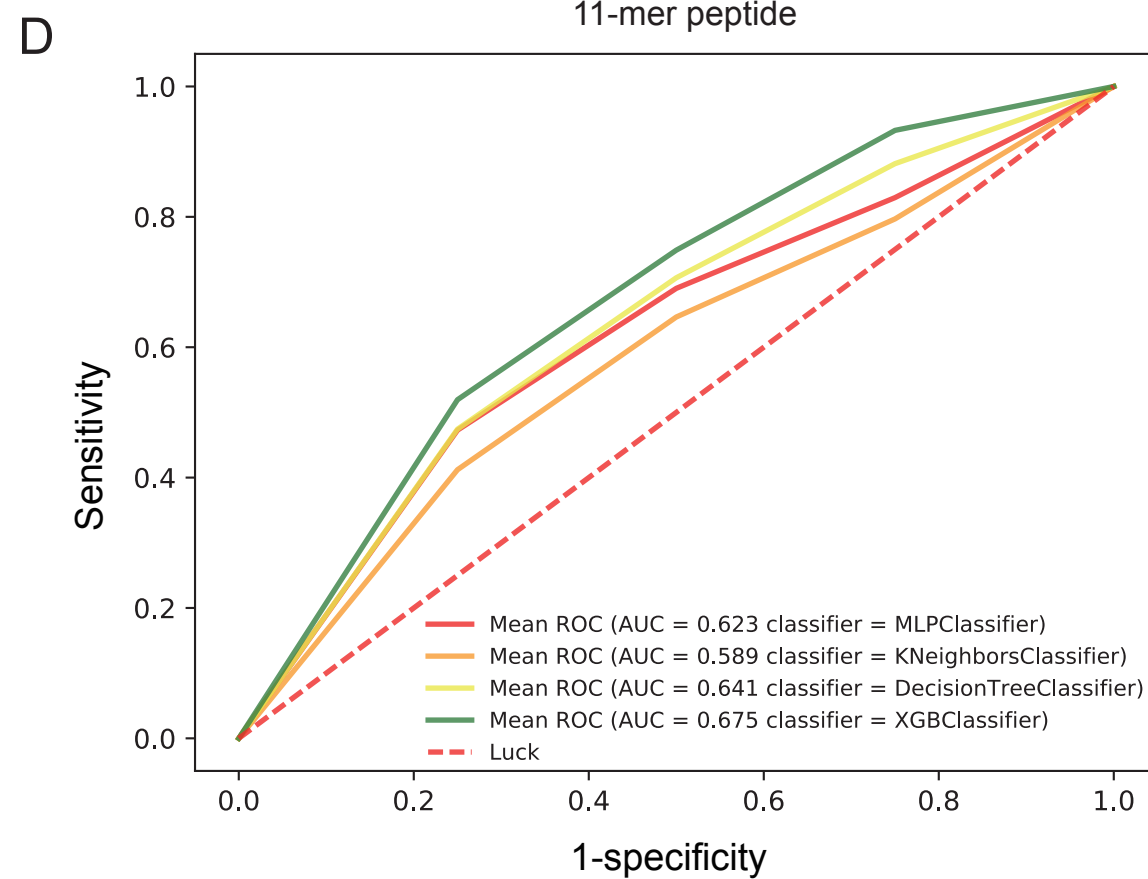

Supplement: Supplementary file 1 — Additional file 1: Figure S1. A. Pipeline of calculating hydrophobicity immunogenicity score. B-D. Performance of three models corresponding to 9mer, 10 mer and 11mer peptides. [file 13073_2019_679_MOESM1_ESM.pdf]
